# Transcriptome Concordance Between Borderline Tumors and Endometrioid Carcinoma: An Integrative Genomic Analysis

**DOI:** 10.1002/cam4.70601

**Published:** 2025-01-22

**Authors:** Mio Takahashi, Kohei Nakamura, Tatsuyuki Chiyoda, Chihiro Okada, Sachio Nohara, Reika Takamatsu, Shintaro Yanazume, Hiroaki Kobayashi, Hiroshi Nishihara, Wataru Yamagami

**Affiliations:** ^1^ Department of Obstetrics and Gynecology Keio University School of Medicine Tokyo Japan; ^2^ Center for Cancer Genomics Keio University School of Medicine Tokyo Japan; ^3^ Department of Biomedical Informatics, Communication Engineering Center, Electronic Systems Business Group Mitsubishi Electric Software co. Ltd Amagasaki Hyogo Japan; ^4^ Department of Obstetrics and Gynecology, Faculty of Medicine Kagoshima University, Kagoshima Kagoshima Japan

**Keywords:** borderline ovarian tumor, genomic analysis, ovarian cancer, RNA sequencing, transcriptome

## Abstract

**Background:**

Borderline ovarian tumors (BOTs) differ from ovarian carcinomas in their clinical presentation and behavior, yet their molecular characteristics remain poorly understood. This study aims to address this gap by integrating whole‐exome sequencing (WES) and RNA sequencing (RNA‐seq) to compare BOTs with high‐grade serous carcinoma (HGSC), endometrioid carcinoma (EC), and clear‐cell carcinoma (CCC).

**Objective:**

To elucidate the molecular features of BOTs and evaluate their similarities and differences in comparison to HGSC, EC, and CCC.

**Methods:**

The study analyzed 44 ovarian tumor samples, employing WES to identify genomic alterations and RNA‐seq to examine transcriptomic profiles. Comparative analyses were conducted to investigate the molecular relationships among the tumor types.

**Results:**

The genomic analysis revealed that BOTs share significant similarities with EC. Furthermore, the transcriptomic data highlighted a novel and substantial similarity between BOTs and EC, suggesting deeper biological linkages, including potentially shared oncogenic pathways or tumor microenvironmental factors. These findings challenge traditional classifications and suggest a closer molecular alignment of BOTs with EC than previously understood.

**Conclusions:**

This study provides new insights into the molecular characteristics of BOTs, demonstrating their significant resemblance to EC at both the genomic and transcriptomic levels. These results underscore the potential need to reconsider the molecular classification of ovarian tumors and open new avenues for research into the pathogenesis and treatment strategies for BOTs.

## Graphical abstract

This study represents the first comprehensive integration of whole‐exome and RNA sequencing to compare the molecular profiles of borderline ovarian tumors (BOTs) with high‐grade serous carcinoma, endometrioid carcinoma (EC), and clear‐cell carcinoma. Our findings reveal notable genomic and transcriptomic similarities between BOTs and ECs, suggesting shared oncogenic pathways. These insights could reshape the molecular classification of ovarian tumors and influence future therapeutic strategies.

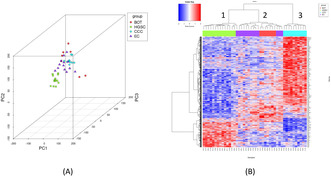

**Abbreviations**
BOT: borderline ovarian tumor
CCC: clear‐cell carcinoma
EBOT: endometrioid borderline ovarian tumor
EC: endometrioid carcinoma
HGSC: high‐grade serous carcinoma
MBOT: mucinous borderline ovarian tumor
SBOT: serous borderline ovarian tumor
SMBOT: seromucinous borderline ovarian tumor

## 1 Introduction

Although borderline ovarian tumors (BOTs), which tend to occur in young women, are noninvasive, they may occasionally become malignant [1, 2]. Approximately, 2000 women are diagnosed with BOTs in Japan annually [3], and BOTs account for 10%–15% of epithelial ovarian tumors [4]. BOTs are classified into several histological types: serous BOTs (SBOTs), mucinous BOTs (MBOTs), endometrioid BOTs (EBOTs), clear‐cell BOTs, and seromucinous BOTs (SMBOTs). The histological criteria for the diagnosis of BOTs include nuclear atypia, stratification of the epithelium, formation of microscopic papillary projections, and absence of stromal invasion [4]. While evidence regarding the prevention of BOTs using oral contraceptives is not as definitive as the case for ovarian cancer, BOTs are more frequent in women with a history of infertility [5]. In contrast, a case–controlled study on BOTs conducted in Italy [6] reported a lower risk of BOTs in oral conceptive users and higher risk in women reporting late age at first birth; moreover, the risk factors associated with ovarian cancer were similar to those associated with BOTs. Currently, the prognosis of BOTs is relatively good [2]. Most BOTs in early stages have a good prognosis. Link et al. described factors such as aneuploidy, microinvasive disease, residual disease after primary surgery, cytologic atypia, and a high mitotic index as being associated with a poor prognosis. Patients with these characteristics are at risk of poor prognosis, even at low stages [2].

The efficacy of conventional chemotherapy on BOTs is limited. This limitation is due to the low proliferation rates of BOTs, which contribute to their poor response to cytotoxic chemotherapy. Some researchers are aiming to identify mutations associated with the early stages of BOTs and develop effective post‐surgical therapies. Recent studies have suggested several hypotheses for the tumorigenesis of BOTs, including the incessant ovulation, gonadotropin hormone, and inflammation hypotheses [7, 8, 9, 10]. Previous studies on BOTs and their first two most common subtypes, SBOTs and MBOTs, have shown that mutations in *KRAS, BRAF*, and *ERBB2*, and overexpression of p53 and claudin‐1 characterized SBOTs, and that *KRAS* mutation, *ERBB2* mutation or amplification, strong trefoil factor‐3 (TFF3) expression, and HER‐2/neu amplification accounted for the occurrence of a certain proportion of MBOTs [7, 8, 9, 10]. Therefore, detecting the status of *KRAS, ERBB2, P53*, or *BRAF* mutations may be useful for predicting or investigating the possibility and tendency for the recurrence of BOTs or invasive ovarian carcinoma in well‐controlled clinical settings.

Several preclinical studies have reported the activation of pathways such as mitogen‐activated protein kinase (MAPK)/extracellular signal‐regulated kinase (ERK), phosphatidylinositol 3‐kinase (PI3K)/AKT/mTOR, Hedgehog, and angiogenesis in both SBOTs and MBOTs, suggesting potential targets for innovative therapies [1, 11, 12, 13, 14, 15, 16, 17, 18, 19, 20]. Clinical trials focusing on these pathways are ongoing; however, conclusive results are awaited as the research in this field is still in the nascent stage [21, 22, 23].

The pathogenesis and molecular mechanisms underlying BOTs have not yet been completely elucidated. Surgical intervention remains the cornerstone of treatment for this disease, and the diagnosis is mainly based on histopathological analysis. Although modern molecular biology techniques, including whole‐exome sequencing (WES) and gene expression profiling, have provided insights into the pathogenesis of BOTs, an integrative genomic analysis comparing BOTs and ovarian cancers is lacking. Here, we performed a comprehensive genome and transcriptome analysis, integrating WES and RNA‐sequencing (RNA‐seq) data for seven BOTs and 37 primary ovarian carcinoma samples. To the best of our knowledge, thus far, omics‐wide integrated analyses have not been performed for cohorts containing samples from patients with BOTs. Therefore, we focused on the molecular characteristics of BOTs and ovarian carcinoma. Large‐scale identification of the molecular features of BOTs may provide important insights regarding the key molecular characteristics associated with ovarian carcinoma and differences between histotypes and may contribute to improvement in the treatment strategies for BOTs.

## 2 Materials and Methods

### 2.1 Patient Selection

This study was part of a research project approved by the Ethics Committee of the Keio University School of Medicine (Japan, Tokyo; Approval Number: 20190111). All procedures involving human participants were performed in accordance with the ethical standards of the institutional and/or national research committee or both. This study conformed to the 1964 Declaration of Helsinki and its later amendments or comparable ethical standards. Written informed consent was obtained from each participant before inclusion in the study.

All patients diagnosed with ovarian tumors who provided written informed consent were included in the study. This group comprised 44 patients who underwent WES: 14 with high‐grade serous carcinoma (HGSC), 13 with endometrioid carcinoma (EC), 10 with clear‐cell carcinoma (CCC), and seven with BOTs, including SBOTs (*n* = 4), SMBOTs (*n* = 1), MBOTs (*n* = 1), and EBOTs (*n* = 1) (Table S1). The diagnosis was confirmed by two expert pathologists based on histological and clinical data. No specific exclusion criteria were set; all patients who met the inclusion criteria were analyzed.

### 2.2 DNA Extraction

Tissue samples collected from patients who underwent surgery were fixed using the *PAX* gene tissue system (Qiagen, Germantown, MD, USA) and embedded in paraffin. A pathologist evaluated the tumor cell content by examining hematoxylin–eosin‐stained slides, which were macrodissected when required. Genomic DNA was quantified using a Qubit4 fluorometer (Q33236, Thermo Fisher Scientific, Waltham, MA, USA), and its quality was assessed using the DNA integrity number (DIN) score calculated using the Agilent 4150 TapeStation (Agilent Technologies, Waldbronn, Germany). The minimum amount of DNA extracted was 150 ng. DNA libraries were prepared for genome sequencing when the quality of DNA had a DIN score above 2.0.

### 2.3 WES

Whole‐exome libraries were prepared using xGen Exome Research Panel v2 (Integrated DNA Technologies Inc., Coralville, IA, USA) and sequenced using the NovaSeq 6000 System (Illumina, San Diego, CA, USA) in the 150‐bp paired‐end mode. Genome annotation and curation for analyzing sequencing data were performed using an original bioinformatics pipeline created on GenomeJack (Mitsubishi Electric Software Co. Ltd., Tokyo, Japan; http://genomejack.net/english/index.html). In this pipeline, paired reads with low sequence quality were discarded, and the next‐generation sequencing reads were mapped to the human reference genome (UCSC human genome 19) using the Illumina DRAGEN Bio‐IT platform v3.8, resulting in a mean depth of 150 × −250×. To identify single‐nucleotide variants (SNVs), SAMtools v1.9 was used to process the sequencing reads, and defective SNVs showing conflict between pairwise reads were discarded [24]. SAMtools mpileup and VarScan [25] v2.4.0 mpileup2snp/mpileup2indel were used to identify the variants. For determining statistical significance, the *P*‐value obtained using Fisher's exact test in VarScan calling was set to 0.01.

Alterations in cancer‐specific somatic genes, such as SNVs, indels, and copy number alterations, were identified. Tumor mutation burden (TMB) was defined as the number of nonsynonymous variants (excluding indels) in the entire region. TMB‐H tumors were defined as those with at least 200 nonsynonymous variants. All detected gene alterations in 728 census genes (COSMIC v87) were annotated and curated using the COSMIC (https://cancer.sanger.ac.uk/cosmic), ClinVar (https://www.ncbi.nlm.nih.gov/clinvar/), CIViC (https://civicdb.org/home), SnpEff4.2, and Clinical Knowledge Base (CKB) (https://ckb.jax.org/) databases.

### 2.4 RNA Sequencing

Total RNA was extracted from tissue samples using the TRIzol reagent, and RNA integrity and concentration were determined using the Agilent 2100 Bioanalyzer and the Qubit RNA HS Assay Kit, respectively. Samples were used for rRNA depletion using the NEBNext rRNA Depletion Kit (human/mouse/rat). Subsequently, the NEBNext Ultra II Directional RNA Library Prep Kit for Illumina was utilized to prepare sequencing libraries from the rRNA‐depleted RNA. This preparation included steps for first‐ and second‐strand cDNA synthesis, the latter incorporating dUTP for strand specificity, followed by end repair, adapter ligation, and PCR amplification. Prepared libraries were quantified using the Qubit dsDNA HS Assay Kit and assessed for size distribution using the Agilent High Sensitivity DNA Kit. Sequencing was performed on an Illumina platform to achieve at least 30 million paired‐end reads per sample, with a read length of 150 bp.

Raw reads were processed to trim adapters and low‐quality bases, aligned to the reference genome using the STAR aligner, and differential expression analysis was conducted with DESeq2. Genes with an adjusted *p*‐value < 0.05 and a fold change > 2 were deemed significantly differentially expressed. To control for multiple hypothesis testing and to reduce the false discovery rate in the post hoc analysis, Tukey's honest significant difference test with Holm's adjustment was applied, considering that *q*‐values < 0.001 indicated highly significant differential expression between groups. Furthermore, to elucidate the complex patterns of gene expression and to identify coexpression modules, bidirectional hierarchical clustering was performed on significantly expressed genes. This was visualized using heatmaps, with genes and samples hierarchically clustered based on their expression profiles to reveal groups with similar expression patterns.

## 3 Results

### 3.1 Patient Characteristics

In our analysis, we included patients with BOTs. Sequencing was performed on 44 ovarian tumor samples, comprising HGSCs (*n* = 14), ECs (*n* = 13), CCCs (*n* = 10), and BOTs (*n* = 7). The seven BOT samples were histologically classified into SBOTs (*n* = 4), SMBOTs (*n* = 1), MBOTs (*n* = 1), and EBOTs (*n* = 1). According to the International Federation of Gynecology and Obstetrics (FIGO) 2014 staging system, all BOT cases were staged as IA. The median age at diagnosis was 48 years (range: 36–67 years).

### 3.2 Mutational Landscape

WES was performed on a diverse cohort of ovarian tumor samples, revealing a spectrum of genomic alterations. Twenty genes frequently exhibiting mutations were selected for a more detailed examination. The most prevalent mutations in HGSC were in *TP53* (detected in 100% of cases), followed by *BRCA1* and *BRCA2* mutations in 36% and 14% of cases, respectively. Notably, among cases harboring *BRCA1/2* mutations, three *BRCA1* and one *BRCA2* mutation were determined to be germline, all within HGSC samples, fitting the criteria for hereditary breast and ovarian cancer (HBOC) syndrome. CCCs exhibited a high frequency of *ARID1A* (70%) and *PIK3CA* (50%) mutations, with *KRAS* mutations present in 30% of the samples. ECs showed a diverse mutational profile with significant occurrences of mutations in *ARID1A* (54%), *PTEN* (46%), and *PIK3CA* (46%). Two cases of EC also exhibited somatic *MSH6* mutations, which were associated with TMB‐H and MSI‐H. The loss of MSH6 expression, observed through immunohistochemistry, was consistent with these genomic findings, indicating a mismatch repair (MMR) deficiency. BOTs most commonly harbored mutations in *KRAS* (43%) and *BRAF* (57%). Additional mutations in *ARID1A*, *PIK3CA, CTNNB1, PTEN*, and *MSH2* occurred at a frequency of 14%. An MBOT case presented with concurrent mutations in *KRAS* and *BRAF*. While no cases of BOTs displayed mutations in *BRCA1/2* or *TP53*, one case harbored a germline *MSH2* mutation, leading to a diagnosis of Lynch syndrome. However, this case did not exhibit TMB‐H or MSI‐H, and MMR proteins were intact upon immunohistochemistry evaluation, suggesting that the germline *MSH2* variant may not contribute to the pathogenesis of BOTs. Characteristics of patients with BOT and genetic profile were summarized in Table 1.

**TABLE 1:** Characteristics of patients with borderline ovarian tumor and genetic profile.

No	Age	Stage	Histological type	TMB (nonsynonymous SNVs/sample)	MSI‐status	Actionable gene alterations	Copy number alterations
1	52	I	Serous borderline tumor	94	MS‐stable	*BRAF* p.V600E	None
2	45	I	Serous borderline tumor	69	MS‐stable	*KRAS* p.G12V, *PIK3CA* p.Q546R, *ARID1A* p.L2238Rfs*30	None
3	48	I	Serous borderline tumor	67	MS‐stable	*BRAF* p.V600E	None
4	36	I	Serous borderline tumor	56	MS‐stable	Germline *MSH2* p.H639Y, *KRAS* p.G12V, *PTEN* p.S229*	None
5	57	I	Seromucinous borderline tumor	56	MS‐stable	*BRAF* p.V600E, *CDKN2A* p.S56Tfs*89	None
6	67	I	Mucinous borderline tumor	57	MS‐stable	*KRAS* p.G12D, *BRAF* p.G469A	None
7	42	I	Endometrioid borderline tumor	67	MS‐stable	*CTNNB1* p.S37F, *ARID1A* p.Q372Sfs*19	None

**FIGURE 1:**
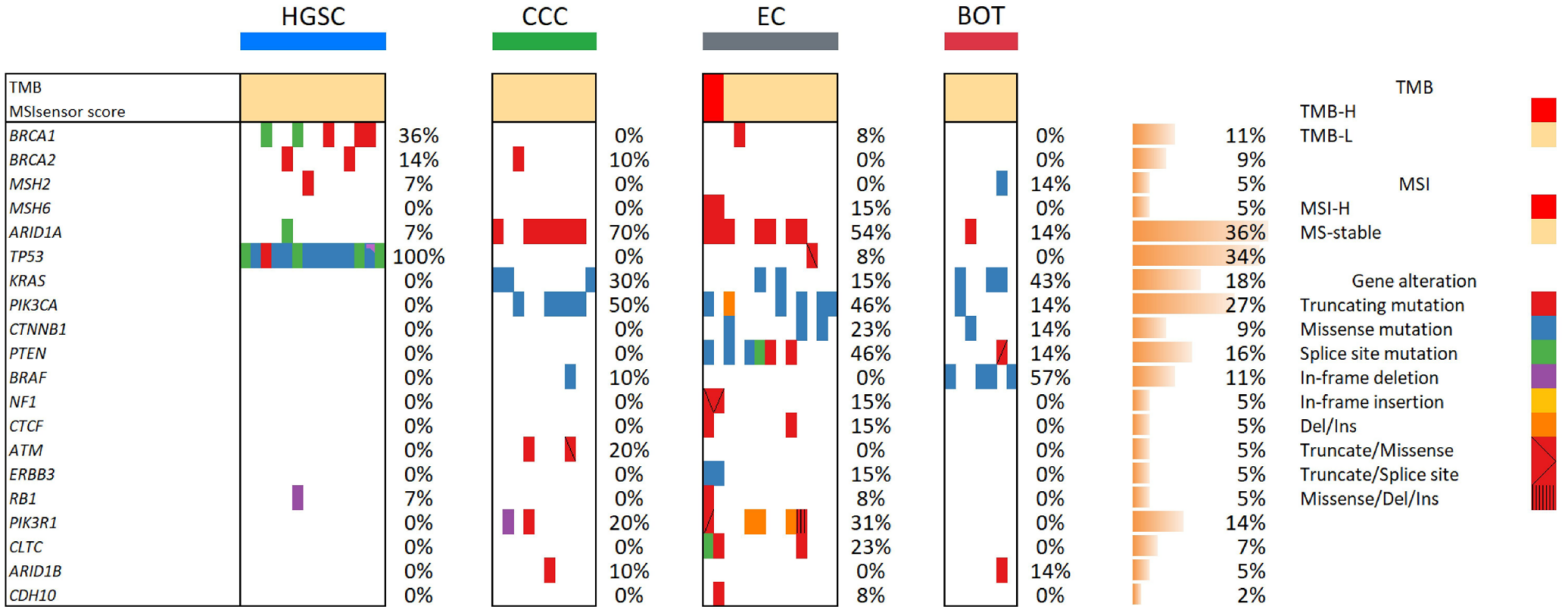
Actionable gene variants identified in ovarian carcinomas and BOTs. This figure displays a heatmap summarizing the genomic analysis from the WES analysis of 44 patients. It compares the tumor mutational burden, microsatellite instability, and pathogenic mutations across various ovarian cancer histotypes and BOTs.

These findings are presented as a heatmap detailing the mutational frequencies across the four histotypes for the selected 20 genes (Figure 1).

### 3.3 RNA‐Seq

**FIGURE 2:**
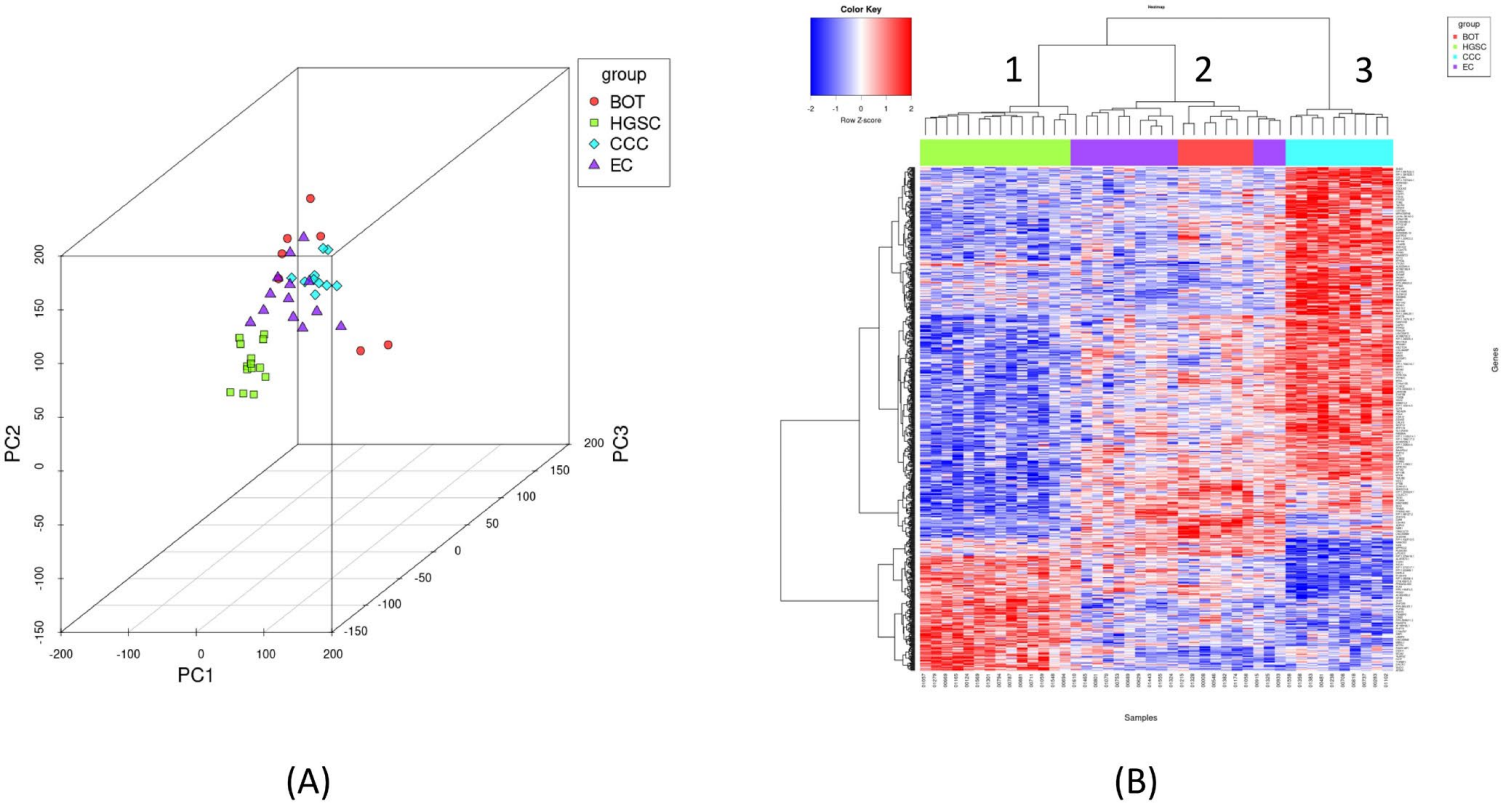
Principal component analysis and expression heatmap of ovarian tumors. (A) 3D plots of the first principal component (PC1) versus the second principal component (PC2) versus the third principal component (PC3) based on RNA sequences of 47 OC tumors. Each point represents a tumor that is colored based on histotype. Figure 1A depicts the tumors of the same histology clustering together. (B) RNA expression analysis. Heatmap displaying expression patterns for the 723 most variable transcripts across the patient cohort (*n* = 44). The RNA‐seq raw counts (log2 scale) of CC, EC, HGSC, and BOT samples in our cohort were compared. An expression value of *q*‐value < 0.05 was set for overexpression (red) and underexpression (blue).

We performed comprehensive RNA‐seq analysis on 44 ovarian tumor samples of various histotypes (HGSC, EC, CCC, and BOTs), resulting in 22,234 Ensembl genes suitable for differential expression analyses. To determine the extent to which the HGSC, EC, CC, and BOT histotypes clustered separately based on gene expression, principal component analysis was performed; the results of which are presented in Figure 2A.

Hierarchical clustering, using the 31,729 transcripts with the highest variance across the cohort, showed that the patients were clustered into three main clusters (Clusters 1, 2, and 3) (Figure 2B): Clusters 1 and 3 were mainly composed of HGSC and CCC, respectively, whereas Cluster 2 contained both EC and BOTs. Samples classified as HGSC clustered together in Cluster 1. Samples classified as CCC clustered together in Cluster 3. The samples classified as EC clustered together, except for one sample in Cluster 1, which was EC Grade 3, indicating that this phenotype was similar to that of HGSC. Samples classified as BOTs were present in Cluster 2, which mainly comprised EC.

Differential expression analysis identified the highest number of differentially expressed genes (DEGs) between HGSC and CCC, and the lowest number of DEGs between EC and BOTs (Benjamini–Hochberg adjusted *P‐*value < 0.05). Notably, we identified 546 DEGs (125 overexpressed and 421 underexpressed) between HGSC and CCC, 95 DEGs (44 overexpressed and 51 underexpressed) between HGSC and EC, 108 DEGs (37 overexpressed and 71 underexpressed) between HGSC and BOTs, 151 DEGs (27 overexpressed and 124 underexpressed) between EC and CCC, two DEGs (one overexpressed and one underexpressed) between EC and BOTs, and 87 DEGs (62 overexpressed and 25 underexpressed) between CCC and BOTs. Gene Ontology enrichment analysis could not be performed as we identified only two DEGs between ECs and BOTs.

**FIGURE 3:**
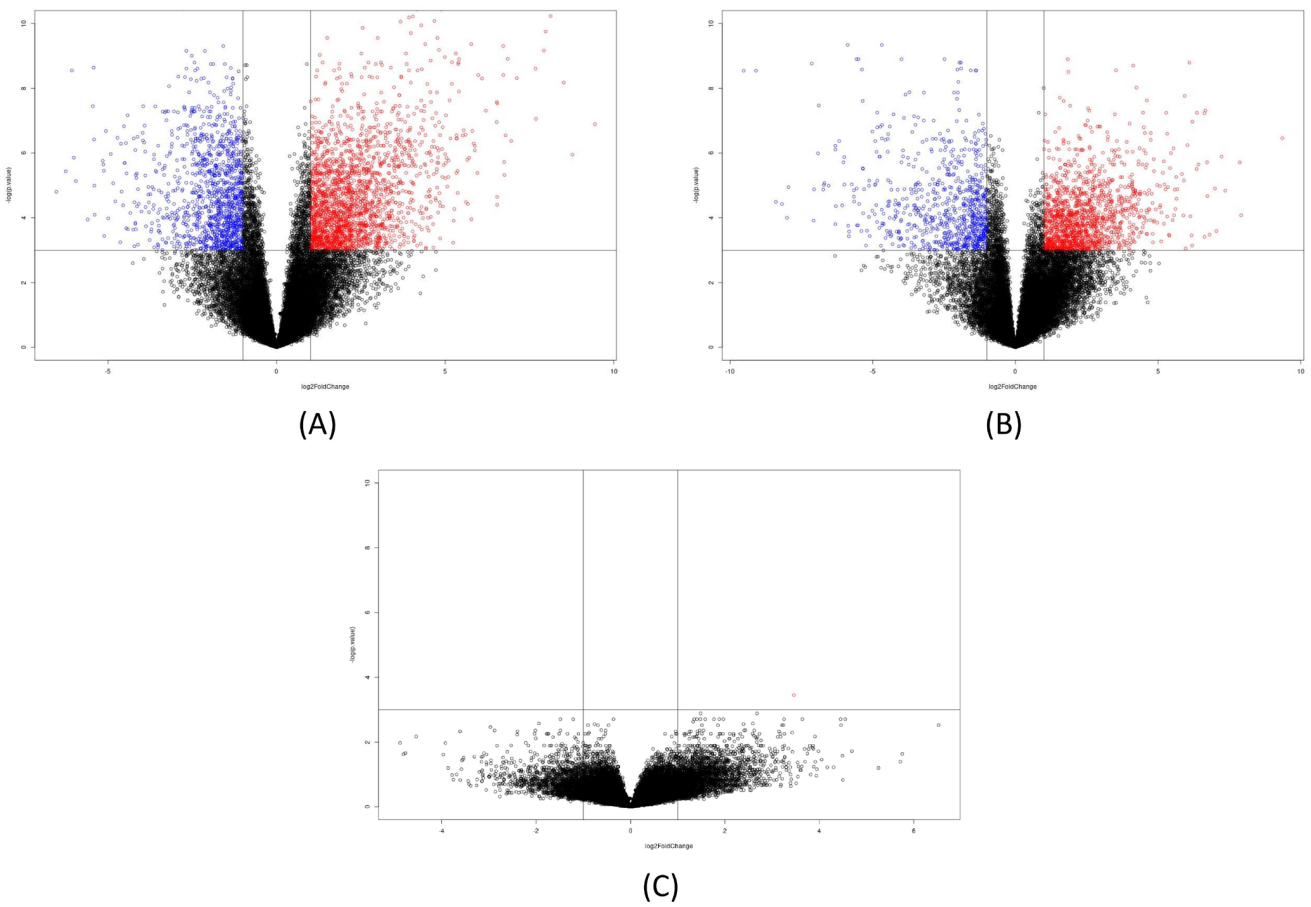
Volcano plots of differential gene expression in borderline ovarian tumors compared to other histotypes. (A) BOTs and HGSC, (B) BOTs and CC, and (C) BOTs and EC. The plot reports the negative log10 of the Benjamini–Hochberg adjusted *P*‐value against the log2 fold‐change of the RNA‐seq expression counts. The significance level for gene regulation was set to 0.05. Each gene is represented by a dot; these dots are highlighted in blue to indicate the downregulated genes in BOTs, red to indicate the upregulated genes in BOTs, and gray to indicate the unregulated genes.

Moreover, we explored the differential gene expression between the BOT histotype and the other three ovarian cancer histotypes (HGSC, CCC, and EC) by constructing volcano plots to visualize the magnitude of expression differences and statistical significance of these changes (Figure 3A–C).

## 4 Discussion

While previous studies have identified genomic similarities between BOTs and other ovarian cancer types, including common mutations in *KRAS* and *BRAF* [7, 8, 9, 10], the transcriptomic parallels we observed between BOTs and ECs represent a novel finding. This groundbreaking transcriptomic resemblance, as demonstrated by our comprehensive RNA‐seq analysis, provides new insights into the molecular biology of BOTs that had not been previously understood. The molecular echo between BOTs and EC at the transcriptomic level, a first‐time report in the literature, underscores a potentially deeper biological linkage that could influence both diagnostic and therapeutic strategies, setting our study apart from those cited earlier [7, 8, 9, 10].

Given this innovative revelation, investigating BOTs is critical not only in terms of their histological and genomic characteristics but also their transcriptomic profiles. This alignment at the RNA level might indicate shared oncogenic pathways or tumor microenvironments that are not apparent at the DNA level alone, suggesting potential therapeutic overlaps with EC treatments that target these molecular pathways [1, 11, 12, 13, 14, 15, 16, 17, 18, 19, 20].

Furthermore, considering the genomic data obtained from WES, we hypothesize that BOTs and EC may resemble each other not only in their molecular profiles but potentially in clinical features such as tumor origin or malignancy. This observation is particularly noteworthy because EC is commonly associated with endometriosis, whereas the origin of BOTs remains elusive. Notably, the transcriptomic similarity does not extend to CCC, which is distinctly different from EC in its gene expression profile. This divergence between BOTs and CCC at the transcriptomic level highlights the unique position of BOTs in the spectrum of ovarian neoplasms and underscores the complexity of ovarian tumor classification based on molecular biology.

This study, while comprehensive, has some limitations. First, the sample size of BOTs was relatively small, and the tumors collected were highly heterogeneous, both between and within tumor types. For instance, the absence of molecular stratification of ECs limits our ability to identify potential subtype‐specific transcriptomic patterns, which could enhance the interpretation of our findings. A larger cohort of BOT samples would be necessary to validate these preliminary results and ensure their applicability to broader patient populations. Additionally, the cross‐sectional nature of our study limits our ability to draw definitive conclusions about the clinical progression and long‐term outcomes associated with the transcriptomic similarities observed. Longitudinal studies tracking the clinical outcomes of patients with BOTs compared with those of patients with EC are needed to understand the implications of these molecular similarities on patient management and prognosis.

A previous retrospective study reported that systematic lymph node dissection for apparent early‐stage low‐grade EC did not substantially improve patient staging or prognosis [11]. In our study, all other EC cases, except for one case of G3 EC, were low‐grade EC cases. The reason underlying the similarity between the transcriptomes of BOTs and EC might be associated with tumor malignancy, although this warrants further investigations. This potential linkage between transcriptomic profiles and tumor behavior emphasizes the need for more comprehensive studies to explore whether these similarities might impact treatment decisions or prognostic assessments.

The distinct yet overlapping molecular profiles visualized in our hierarchical clustering analyses not only support the genomic data but also highlight the novel transcriptomic similarity to EC. This finding could lead to a reevaluation of how BOTs are classified and treated, advocating for a more nuanced approach that incorporates transcriptomic data into clinical decision‐making processes.

Furthermore, the clinical implications of these transcriptomic findings are profound. The potential sharing of functional pathways between BOTs and ECs, as indicated by their similar gene expression profiles, suggests a possible risk of aggressive behavior in certain cases of BOTs that could otherwise be underestimated and undertreated. Further research should be conducted to explore these shared transcriptomic features in large cohorts; this will help validate our findings and may aid the adjustment of treatment protocols to accurately reflect the underlying molecular biology [8, 9, 10].

In conclusion, our findings not only reiterate the known genomic similarities between BOTs and ECs but also introduce a critical new dimension of transcriptomic similarity, opening avenues for further research and potential shifts in therapeutic strategies. We believe that our findings substantially contribute to the field by proposing a paradigm shift in how BOTs are perceived and managed clinically.

## Author Contributions

**Mio Takahashi:** conceptualization (equal), resources (equal), writing – original draft (equal). **Kohei Nakamura:** conceptualization (equal), data curation (equal), funding acquisition (equal), resources (equal), supervision (equal), writing – original draft (equal), writing – review and editing (equal). **Tatsuyuki Chiyoda:** resources (equal). **Chihiro Okada:** data curation (equal). **Sachio Nohara:** data curation (equal). **Reika Takamatsu:** resources (equal). **Shintaro Yanazume:** resources (equal). **Hiroaki Kobayashi:** resources (equal). **Hiroshi Nishihara:** conceptualization (equal). **Wataru Yamagami:** conceptualization (equal), resources (equal), supervision (equal).

## Disclosure

All authors declare no conflicts of interest.

## Ethics Statement

Approval of the research protocol by an Institutional Reviewer Board: This study was part of a research project approved by the ethics committee of the Keio University School of Medicine (Approval Number: 20190111). This study conformed to the 1964 Declaration of Helsinki and its later amendments or comparable ethical standards.

## Consent

Informed consent was obtained from each participant before inclusion in the study.

## Conflicts of Interest

The authors declare no conflicts of interest.

## Supporting information


**Table S1.** Characteristics of patients with high‐grade serous, endometrioid, clear‐cell ovarian carcinoma, and borderline ovarian tumor.

## Data Availability

The data supporting the findings of this study are not available as the study participants did not consent to the public sharing of their data.

## References

[1] J. Boyd , B. Luo , S. Peri , et al., “Whole Exome Sequence Analysis of Serous Borderline Tumors of the Ovary,” Gynecologic Oncology 130, no. 3 (2013): 560–564, 10.1016/j.ygyno.2013.06.007. 23774303 PMC4083840

[2] C. J. Link , E. Reed , G. Sarosy , and E. C. Kohn , “Borderline Ovarian Tumors,” American Journal of Medicine 101, no. 2 (1996): 217–225, 10.1016/S0002-9343(96)80079-9. 8757363

[3] H. Tokunaga , M. Mikami , S. Nagase , et al., “The 2020 Japan Society of Gynecologic Oncology guidelines for the treatment of ovarian cancer, fallopian tube cancer, and primary peritoneal cancer,” Journal of Gynecologic Oncology 32 (2021): e49, 10.3802/jgo.2021.32.e49. 33650343 PMC7930451

[4] D. M. Gershenson , “Management of Borderline Ovarian Tumours,” Best Practice & Research. Clinical Obstetrics & Gynaecology 41 (2017): 49–59, 10.1016/j.bpobgyn.2016.09.012. 27780698

[5] R. Harris , A. S. Whittemore , and J. Itnyre , “Characteristics Relating to Ovarian Cancer Risk: Collaborative Analysis of 12 US Case‐Control Studies. III. Epithelial Tumors of Low Malignant Potential in White Women. Collaborative Ovarian Cancer Group,” American Journal of Epidemiology 136, no. 10 (1992): 1204–1211, 10.1093/oxfordjournals.aje.a116428. 1476142

[6] F. Parazzini , C. Restelli , C. La Vecchia , et al., “Risk Factors for Epithelial Ovarian Tumours of Borderline Malignancy,” International Journal of Epidemiology 20, no. 4 (1991): 871–877, 10.1093/ije/20.4.871. 1800425

[7] M. F. Fathalla , “Incessant Ovulation—A Factor in Ovarian Neoplasia?,” Lancet 298, no. 7716 (1971): 163, 10.1016/s0140-6736(71)92335-x. 4104488

[8] F. E. Van Leeuwen , H. Klip , T. M. Mooij , et al., “Risk of Borderline and Invasive Ovarian Tumours After Ovarian Stimulation for In Vitro Fertilization in a Large Dutch Cohort,” Human Reproduction 26, no. 12 (2011): 3456–3465, 10.1093/humrep/der322. 22031719 PMC3212878

[9] T. Riman , P. W. Dickman , S. Nilsson , et al., “Risk Factors for Epithelial Borderline Ovarian Tumors: Results of a Swedish Case–Control Study,” Gynecologic Oncology 83, no. 3 (2001): 575–585, 10.1006/gyno.2001.6451. 11733975

[10] R. J. Kurman , L. H. Ellenson , and B. M. Ronnett , Blaustein's Pathology of the Female Genital Tract, 6th ed. (Germany: Springer Science and Business Media, 2011), 529–578, 10.1007/978-1-4419-0489-8_13.

[11] Y. Sun , J. Xu , and X. Jia , “The Diagnosis, Treatment, Prognosis and Molecular Pathology of Borderline Ovarian Tumors: Current Status and Perspectives,” Cancer Management and Research 12 (2020): 3651–3659, 10.2147/CMAR.S250394. 32547202 PMC7246309

[12] M. S. Anglesio , J. M. Arnold , J. George , et al., “Mutation of ERBB2 Provides a Novel Alternative Mechanism for the Ubiquitous Activation of RAS‐MAPK in Ovarian Serous Low Malignant Potential Tumors,” Molecular Cancer Research 6, no. 11 (2008): 1678–1690, 10.1158/1541-7786.MCR-08-0193. 19010816 PMC6953412

[13] R. Mackenzie , S. Kommoss , B. J. Winterhoff , et al., “Targeted Deep Sequencing of Mucinous Ovarian Tumors Reveals Multiple Overlapping RAS‐Pathway Activating Mutations in Borderline and Cancerous Neoplasms,” BMC Cancer 15 (2015): 415, 10.1186/s12885-015-1421-8. 25986173 PMC4494777

[14] A. Malpica and K. K. Wong , “The Molecular Pathology of Ovarian Serous Borderline Tumors,” Annals of Oncology 27 (2016): i16–i19, 10.1093/annonc/mdw089. 27141064 PMC4852276

[15] P. Ozretíc , D. Trnski , V. Musani , et al., “Non‐canonical Hedgehog Signaling Activation in Ovarian Borderline Tumors and Ovarian Carcinomas,” International Journal of Oncology 51, no. 6 (2017): 1869–1877, 10.3892/ijo.2017.4156. 29039491

[16] R. N. Grisham , G. Iyer , E. Sala , et al., “Bevacizumab Shows Activity in Patients With Low‐Grade Serous Ovarian and Primary Peritoneal Cancer,” International Journal of Gynecological Cancer 24, no. 6 (2014): 1010–1014, 10.1097/IGC.0000000000000190. 24978709 PMC4401424

[17] Z. Lu , F. Lin , T. Li , et al., “Identification of Clinical and Molecular Features of Recurrent Serous Borderline Ovarian Tumour,” EClinicalMedicine 46 (2022): 101377, 10.1016/j.eclinm.2022.101377. 35434581 PMC9011028

[18] E. W. Curry , E. A. Stronach , N. R. Rama , et al., “Molecular Subtypes of Serous Borderline Ovarian Tumor Show Distinct Expression Patterns of Benign Tumor and Malignant Tumor‐Associated Signatures,” Modern Pathology 27, no. 3 (2014): 433–442, 10.1038/modpathol.2013.130. 23948749

[19] R. C. Wu , S. J. Chen , H. C. Chen , et al., “Comprehensive Genomic Profiling Reveals Ubiquitous KRAS Mutations and Frequent PIK3CA Mutations in Ovarian Seromucinous Borderline Tumor,” Modern Pathology 33, no. 12 (2020): 2534–2543, 10.1038/s41379-020-0611-3. 32616873

[20] G. L. Ryland , S. M. Hunter , M. A. Doyle , et al., “Mutational Landscape of Mucinous Ovarian Carcinoma and Its Neoplastic Precursors,” Genome Medicine 7 (2015): 87, 10.1186/s13073-015-0210-y. 26257827 PMC4528310

[21] I. M. Shih and R. J. Kurman , “Molecular Pathogenesis of Ovarian Borderline Tumors: New Insights and Old Challenges,” Clinical Cancer Research 11, no. 20 (2005): 7273–7279, 10.1158/1078-0432.CCR-05-0755. 16243797

[22] D. M. Gershenson , “Molecular Targeting of Low‐Grade Serous and Mucinous Carcinomas of the Ovary or Peritoneum,” Translational Cancer Research 4, no. 1 (2015): 29–39, 10.3978/j.issn.2218-676X.2015.01.05.

[23] J. Hirst , J. Crow , and A. Godwin , “Ovarian Cancer Genetics: Subtypes and Risk Factors,” in Ovarian Cancer—From Pathogenesis to Treatment, eds. O. Devaja and A. Papadopoulos (London, United Kingdom: InTech, 2018), 10.5772/intechopen.72705.

[24] S. Li , B. Wang , M. Chang , R. Hou , G. Tian , and L. Tong , “A Novel Algorithm for Detecting Microsatellite Instability Based on Next‐Generation Sequencing Data,” Frontiers in Oncology 12 (2022): 916379, 10.3389/fonc.2022.916379. 35847873 PMC9280483

[25] D. C. Koboldt , Q. Zhang , D. E. Larson , et al., “VarScan 2: Somatic Mutation and Copy Number Alteration Discovery in Cancer by Exome Sequencing,” Genome Research 22, no. 3 (2012): 568–576, 10.1101/gr.129684.111. 22300766 PMC3290792

